# Pattern recognition analyses of brain activation elicited by happy and neutral faces in unipolar and bipolar depression

**DOI:** 10.1111/j.1399-5618.2012.01019.x

**Published:** 2012-06

**Authors:** Janaina Mourão-Miranda, Jorge RC Almeida, Stefanie Hassel, Leticia de Oliveira, Amelia Versace, Andre F Marquand, Joao R Sato, Michael Brammer, Mary L Phillips

**Affiliations:** a Department of Computer Science, Centre for Computational Statistics and Machine Learning, University College LondonLondon, UK; b Department of Neuroimaging, King's College LondonLondon, UK; c Department of Psychiatry, University of Pittsburgh School of MedicinePittsburgh, PA, USA; d Instituto Biomédico, Universidade Federal FluminenseRio de Janeiro; e Center of Mathematics, Computation and Cognition, Universidade Federal do ABCSanto André, Brazil; f Department of Clinical Neuroscience, Institute of Psychiatry, King’s College LondonLondon; g Department of Psychological Medicine, Cardiff UniversityCardiff, UK

**Keywords:** bipolar disorder, depression, fMRI, Gaussian process, patient classification, pattern recognition

## Abstract

**Objectives:**

Recently, pattern recognition approaches have been used to classify patterns of brain activity elicited by sensory or cognitive processes. In the clinical context, these approaches have been mainly applied to classify groups of individuals based on structural magnetic resonance imaging (MRI) data. Only a few studies have applied similar methods to functional MRI (fMRI) data.

**Methods:**

We used a novel analytic framework to examine the extent to which unipolar and bipolar depressed individuals differed on discrimination between patterns of neural activity for happy and neutral faces. We used data from 18 currently depressed individuals with bipolar I disorder (BD) and 18 currently depressed individuals with recurrent unipolar depression (UD), matched on depression severity, age, and illness duration, and 18 age- and gender ratio-matched healthy comparison subjects (HC). fMRI data were analyzed using a general linear model and Gaussian process classifiers.

**Results:**

The accuracy for discriminating between patterns of neural activity for happy versus neutral faces overall was lower in both patient groups relative to HC. The predictive probabilities for intense and mild happy faces were higher in HC than in BD, and for mild happy faces were higher in HC than UD (all p < 0.001). Interestingly, the predictive probability for intense happy faces was significantly higher in UD than BD (p = 0.03).

**Conclusions:**

These results indicate that patterns of whole-brain neural activity to intense happy faces were significantly less distinct from those for neutral faces in BD than in either HC or UD. These findings indicate that pattern recognition approaches can be used to identify abnormal brain activity patterns in patient populations and have promising clinical utility as techniques that can help to discriminate between patients with different psychiatric illnesses.

The absence of biologically relevant diagnostic markers of bipolar disorder results in frequent misdiagnosis of the illness as recurrent unipolar depression in 60% of depressed bipolar individuals seeking treatment (1). This misdiagnosis leads to inadequate treatment that can promote switching to mania and worsen illness outcome (1) in an illness that has a 15% suicide rate and direct annual costs in the USA of $7.6 billion (2). Objective markers of bipolar disorder are therefore needed to aid its accurate diagnosis and distinguish it from unipolar depression as early as possible in affected individuals. Previous studies reported abnormal patterns of subcortical and cortical activity to emotional faces (especially happy faces) in bipolar depressed and unipolar depressed individuals (3–5). A more recent neuroimaging study also indicated that patterns of brain activity and connectivity to emotional stimuli – happy facial expressions – may help to distinguish bipolar and unipolar depressed adults (6). Further studies using different analytic techniques are therefore required to examine the extent to which neuroimaging can accurately help to distinguish between these two diagnoses.

Pattern recognition approaches have been used to classify patterns of neural activity elicited by sensory or cognitive processes (7–10); that is, as *mind-reading* devices that can predict an individual’s brain state. In the clinical context, these approaches have been mainly applied to classify groups of individuals based on brain structural magnetic resonance imaging (MRI) data (11–14). Only a few studies have applied similar methods to functional MRI (fMRI) data (15, 16). For example, these studies reported that healthy subjects and unipolar depressed patients could be discriminated by whole-brain patterns of activity to a specific stimulus (sad faces) (15) or task (verbal working memory) (16). One additional study showed that integrating predictions based on brain activation associated with emotional and affective processing substantially increased the accuracy to discriminate between a heterogeneous group of depressed patients (i.e., patients who were on a variety of medications and with varying degrees of depressive symptoms) and healthy subjects (17).

Direct discrimination between two groups with different psychiatric illnesses using whole-brain activity patterns is much more challenging, however, especially when the symptomatology and underlying neuropathology can be expected to overlap. An additional complication is that it can be difficult to find one task or stimulus that evokes sufficient differential activity between different patient groups. In many situations, group differences are too subtle in relation to the typical noise in fMRI, and they are not easily identified by conventional approaches or even by more sensitive approaches such as pattern recognition. Another interesting question is how different psychiatric illnesses affect discrimination between patterns of neural activity for different stimuli. The discriminative accuracy between patterns of neural activity indicates how stable and different the patterns are. If the patterns are stable but different, then the accuracy for discriminating between them will be above chance level. If the patterns are heterogeneous or overlapping, however, then it is likely that the classification accuracy will be close to chance level. Understanding how psychiatric illnesses with overlapping symptoms affect the within-group pattern discriminability between two different stimuli is the first step to building more complex neural models to ultimately discriminate between patients with different psychiatric illnesses.

In the present study, we aimed to examine the differential effect of unipolar and bipolar depression on the discriminability of patterns of neural activity elicited by happy and neutral facial stimuli. We focused examination on happy and neutral facial stimuli, given previous findings that individuals with bipolar disorder show abnormal neural activity to happy facial expressions in particular.

The analysis framework involved a two-level, novel analytic strategy. In the first level, we applied a Gaussian process classifier (GPC) within each group to discriminate between the brain activity patterns elicited by happy versus neutral stimuli; that is, we computed the within-group classification accuracy for different stimulus contrasts and the predictive probabilities for each stimulus. The predictive probability measures the classifier confidence about the class membership of the test example. In the second level, we used analysis of variance (ANOVA) and post-hoc tests to examine whether the predictive probabilities were significantly different between groups [healthy comparison subjects (HC), currently depressed individuals with recurrent unipolar depression (UD), and currently depressed individuals with bipolar I disorder (BD)]. We also adopted the more conventional approach of examining the ability of the GPC to discriminate between the groups (UD, BD, and HC) using whole-brain activity patterns to each of the three different intensity facial expressions (intense happy, mild happy, and neutral faces).

## Materials and methods

### Participants

We recruited 18 BD and 18 UD based on DSM-IV diagnostic criteria using the Structured Clinical Interview for DSM-IV-TR–Patient edition (SCID-P) (18) for these illnesses (19) {mean age [standard deviation (SD)]: BD = 36 [11] and UD = 32 [9] years; age range: 18–54 years}. The severity of depression was evaluated using the 25-item Hamilton Rating Scale for Depression (25-HDRS) (20) (score > 13). Depressed groups were matched for depression severity (*t* = 1.34, p = 0.18), illness duration (*t* = 0.53, p = 0.59), age at illness onset (*t* = 0.81, p = 0.41), and age (*U* = 122, p = 0.20). Eighteen HC [mean age (SD) = 30 (9)] with no previous psychiatric history (based on SCID-P criteria) or first- or second-degree relatives with a psychiatric history also participated in the study. HC were age- [χ^2^(2) = 3.8, p = 0.15] and gender ratio- [χ^2^(2) = 2.3, p = 0.32] matched with BD and UD (Table 1). All participants were right-handed and had a premorbid IQ higher than 85 (inclusion criterion). Lifetime history and/or current alcohol and illicit substance abuse (determined by saliva and urine screen, respectively) were additional exclusion criteria for HC. While some BD and UD did have a history of comorbid alcohol or substance abuse, as is typical for these populations, BD and UD subjects had been free from alcohol and/or illicit substance abuse or dependence for at least two months before scanning, and had to be free from current alcohol or illicit substances on the day of scanning (determined by saliva and urine screen, respectively). All participants gave informed consent after explanation of the nature and possible consequences of the study. We developed a strategy for measuring total medication load for the patient groups (see *Supplementary material* for a detailed description).

**Table 1:** Demographic and clinical variables

	HC (n = 18)	UD (n = 18)	BD (n = 18)	Statistics	p-value (two-tailed)
Age at scan, years	30 (9)	32 (9)	36 (11)	χ^2^ (2) = 3.8	0.15
Gender (M/F)	2/16	1/17	4/14	χ^2^ (2) = 2.3	0.32
Age of illness onset, years		19 (10)	22 (7)	*t* = 0.81	0.41
Illness duration, years		13 (9)	14 (9)	*t* = 0.53	0.59
Medication load		1.4 (1.1)	2.5 (1.5)	*t* = 2.32	0.02
Lifetime history of alcohol/substance abuse or dependence (Yes/No)		4/14	5/11	χ^2^ (2) = 1.44	0.49
HDRS-25 score		23 (8)	20 (8)	*t* = -1.34	0.18

Values are reported as mean and standard deviation (SD) unless indicated otherwise. BD = currently depressed individuals with bipolar I disorder; HC = healthy comparison subjects; HDRS-25 = 25-item Hamilton Rating Scale for Depression; UD = currently depressed individuals with recurrent unipolar depression.

### Paradigm

**Fig. 1:**
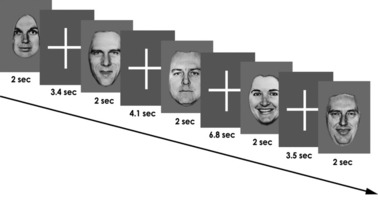
Experimental design. Individuals viewed 20 intense happy expressions (100% happy emotion), 20 mild happy expressions (50% happy emotion), and 20 neutral expressions. Each facial expression was presented for 2 sec, with an inter-stimulus interval (ISI) of variable duration, varied according to a Poisson distribution (mean ISI = 4.9 sec).

All individuals participated in a 6-min event-related experiment. The experiment involved viewing 60 facial expressions from a standardized series of neutral, prototypical, and milder intensity emotion facial expressions, the latter created by morphing neutral and prototypical expressions (21). Individuals viewed 20 intense happy expressions (intense happy emotion), 20 mild happy expressions (50% happy emotion), and 20 neutral expressions. Each facial expression was presented for 2 sec, with an inter-stimulus interval (ISI) of variable duration, varied according to a Poisson distribution (mean ISI = 4.9 sec). Participants labeled the emotion of each face by moving either the index (emotional faces) or middle finger (neutral faces) of the right hand to ensure that attention was directed to the emotional content of the face (Fig. 1).

### Data acquisition

Neuroimaging data were collected using a 3.0 Tesla Siemens Allegra MRI scanner at the University of Pittsburgh/Carnegie Mellon University (CMU) Brain Imaging Research Center (Pittsburgh, PA, USA). Structural three-dimensional sagittal magnetization prepared rapid gradient echo images were acquired in the same session [echo time (TE) = 2.48 msec, repetition time (TR) = 1630 msec, flip angle = 8°, field of view (FOV) = 200 mm, slice thickness = 1 mm, matrix = 256 × 256, 192 continuous slices]. Blood oxygen level-dependent (BOLD) functional images were then acquired with a gradient echo planar imaging (EPI) sequence covering 33 axial slices (3 mm thick, 0 mm gap, TR/TE = 2000/25 msec, FOV = 24 cm, matrix = 64 × 64). All scanning parameters were selected to optimize the BOLD signal quality while maintaining a sufficient number of slices to acquire whole-brain data.

### Data pre-processing

Data were pre-processed using statistical parametric mapping software (SPM5) (http://www.fil.ion.ucl.ac.uk/spm). Data for each participant were first corrected for differences in acquisition time between slices, realigned using the first volume as a reference, and unwarped to correct for static inhomogeneity of the magnetic field and movement by inhomogeneity interactions. Realigned functional images were then co-registered with each participant’s anatomical image and normalized with the anatomical gray matter parameters to the standard Montreal Neurological Institute template, resampled to 3 × 3 × 3 mm^3^ voxels, and spatially smoothed with a Gaussian kernel of 6 mm full-width at half-maximum (FWHM).

### Feature extraction

For each subject, a general linear model (GLM) was constructed in SPM5 with three emotion intensities (neutral, mild happy, intense happy) entered in the design matrix as separate regressors in an event-related design with fixation cross as the baseline. Movement parameters from the realignment stage were entered as covariates of no interest to control for subject movement. Trials were modeled using the canonical hemodynamic response function of SPM5. The images corresponding to the GLM coefficients for each experimental condition (intense happy, mild happy, and neutral) defined the spatial patterns (i.e., vector of features) used as input to the pattern recognition approach.

We used a whole-brain classification approach; that is, there was no feature selection step, which simplifies the analysis pipeline. Feature selection requires a three-way cross-validation procedure (splitting the data into train, test, and validation sets for parameter optimization) to avoid overfitting, which would have been a suboptimal procedure, considering the relatively small sample size in the present study. Maintaining a simple pipeline also improved the translational potential of the method, as a simple technique would be more likely to be incorporated among routine clinical investigations. Whole-brain fMRI-based diagnosis using GLM coefficients has been previously shown to be able to reach around 86% accuracy (15). Furthermore, a recent paper comparing the classification performance of ten different approaches using structural MRI showed that the use of feature selection did not improve the classification performance but substantially increased the computation time (22).

### Pattern classification analysis

We used a GPC, as it provides for each test sample the predictive *probability* of belonging to each class instead of categorical values (e.g., +1 for *Class 1* and –1 for *Class 2*). The predictive probability measures the classifier confidence about the class membership of the test example, and can therefore be used as a proxy for ambiguity or consistency of differential patterns of neural activity (see *Supplementary material* for a detailed description of GPC).

### Within-group stimulus classification

Within each group (HC, BD, and UD) we examined the ability of the GPC to accurately discriminate between the pattern of whole-brain activity for different intensity facial expressions for the following stimulus contrasts: intense happy versus neutral, and mild happy versus neutral. The GPC gave predictive probabilities for stimuli of class 1 and class 2. The predictive probability thereby gave a measure of the confidence of the classifier (i.e., GPC) about the class membership of the test pattern (i.e., the pattern of whole-brain activation for the test subject). For example, if the predictive probability were close to 0.5, the classifier would not be very confident about the class membership of the test pattern of whole-brain activity, thereby indicating that there was not enough information on the pattern of whole-brain activity to discriminate between class 1 and class 2. On the other hand, if the predictive probability were close to one (or zero), the classifier would be very confident about class membership; that is, the whole-brain pattern of neural activity would be consistent with the pattern of neural activity established from the training data and there would be enough information on the pattern to discriminate between class 1 and class 2.

In the within-group analysis, medication load and previous history of substance abuse were included as covariates in the pattern classification analysis using the residual forming matrix framework (see *Supplementary material* for details). Therefore, the between-group comparison, described next, used as variables *predictive probabilities* that had already controlled for the potential confounding effects of medication load and previous substance abuse.

### Between-group comparison of accuracies and predictive probabilities

We used a one-way ANOVA and post-hoc tests (Newman–Keuls) to examine the effect of group upon the predictive probabilities for each of the two stimulus contrasts (intense happy versus neutral and mild happy versus neutral).

### Cross-validation procedure

In each group, we evaluated the performance of GPC to discriminate between neural activity patterns for each of the two different emotion intensities in each of the two stimulus contrasts using a leave-one-subject-out cross-validation test. Here, for each trial, we first used data from all but one individual to train the classifier. Then, we predicted the intensity of facial expressions using the brain scans of the remaining individual (one of each class). We used a threshold of 0.5 to decide its class membership; that is, if the predictive probability were above 0.5 it corresponded to class 1, otherwise (0.5 or less), it corresponded to class 2. The mean classification accuracy was the mean of the true positive (i.e., percentage of examples of class 1 correctly classified) and true negative rates (i.e., percentage of examples of class 2 correctly classified).

The statistical significance of the classifier was determined by permutation testing, as described in the *Supplementary material*.

### Direct group discrimination

We also examined the ability of the GPC to discriminate between the groups (UD, BD, and HC) using whole-brain activity patterns to each of the three different intensity facial expressions (intense happy, mild happy, and neutral faces). For each of the three facial expression intensities, we trained a GPC aiming to directly discriminate between groups for each possible group pair: UD versus HC, BD versus HC, and UD versus BD. We evaluated the performance of the classifiers using a leave-one-pair-out cross-validation test, as employed previously (15). Here, in each trial we first used data from all but one pair of subjects to train the classifier (i.e., we left a matched pair out in each trial). The predictive probability of the remaining two individuals (one in each group) was then computed during the test phase. As previously described, we used a threshold of 0.5 to decide the class membership and computed sensitivity and specificity for each classifier. The statistical significance of the classifier was determined by permutation testing.

## Results

### Within-group stimuli classification

**Fig. 2:**
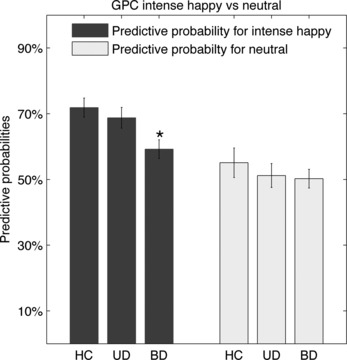
Gaussian process classifier results for the contrast intense happy (i.e., 100% happy) versus neutral expressions. The predictive probability for intense happy faces was significantly lower in currently depressed individuals with bipolar I disorder (BD) than in healthy comparison subjects (HC) (*p = 0.001) and in currently depressed individuals with recurrent unipolar depression (UD) (p = 0.03).

**Fig. 3:**
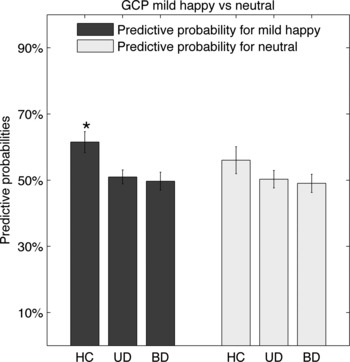
Gaussian process classifier results for the contrast mild happy (i.e., 50% happy) versus neutral. The probability for mild happy was significantly higher in healthy comparison subjects (HC) than in currently depressed individuals with bipolar I disorder (BD) (*p = 0.001) and in currently depressed individuals with recurrent unipolar depression (UD) (*p = 0.001).

The results for the three within-group binary contrasts are presented in Table 2 (accounting for medication load and previous substance abuse as covariates in BD and UD). The predictive probabilities (i.e., the output of the classifier before applying the threshold of 0.5 to decide the pattern’s class membership) for each classifier and each group are also presented in Figures 2 and 3. The GPC accuracies for discriminating between intense happy and neutral faces were significantly above chance level for all groups (BD = 61%; UD = 70%; HC = 81%), but for discriminating between mild happy and neutral faces were significantly above chance only for HC (BD = 47%; UD = 50%; HC = 75%). However, the predictive probabilities for specific stimuli varied across classifier contrasts and across groups.

**Table 2:** Within-group decoding accuracy^a^

Contrast	Accuracy	TP	TN	p-value
**100% happy × neutral**				
HC	0.81	0.94	0.72	0.001
UD	0.70	0.89	0.50	0.002
BD	0.61	0.72	0.50	0.029
**50% happy × neutral**				
HC	0.75	0.83	0.67	0.001
UD	0.50	0.39	0.61	0.589
BD	0.47	0.44	0.50	0.816

BD = currently depressed individuals with bipolar I disorder; HC = healthy comparison subjects; TN = true negative; TP = true positive; UD = currently depressed individuals with recurrent unipolar depression.

a n = 18 for each group.

### Between-group differences in predictive probabilities

#### GPC for intense happy versus neutral faces

A one-way ANOVA revealed a significant main effect of group on the predictive probability for intense happy faces (p = 0.01). Post-hoc tests showed that the mean predictive probability for intense happy faces was significantly higher in HC than BD (p = 0.001), but not HC versus UD. Interestingly, the predictive probability for intense happy faces was significantly higher in UD than BD (p = 0.03). There was no effect of group on the predictive probability for neutral stimuli.

#### GPC for mild happy versus neutral faces

A one-way ANOVA revealed a significant main effect of group on the predictive probability for mild happy faces (p = 0.005). Post-hoc tests showed that the mean predictive probability for mild happy was significantly higher in HC than in BD (p = 0.001) and UD (p = 0.001). There was no significant difference between BD and UD (p > 0.7). There was no effect of group on the predictive probability for neutral stimuli.

### Direct group discrimination

We examined the ability of the GPC to discriminate directly between groups using neural activity patterns elicited for each of the facial expression intensities. The best classification accuracy was obtained when discriminating between BD and UD using patterns of neural activity for mild happy (specificity = 72%, sensitivity = 61%, uncorrected p-value = 0.02), although within-group analyses (above) revealed that both depressed groups had only chance-level accuracies for discriminating between mild happy and neutral faces. However, none of the accuracies was significant after correction for multiple comparisons (see *Supplementary Table 1*).

### Within-group discriminating maps

**Fig. 4:**
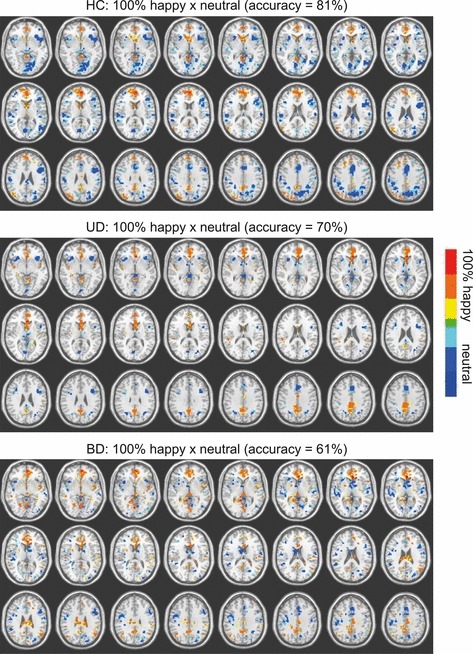
Gaussian process classifier discriminating maps for each group for the contrast of intense happy versus neutral overlaid on an anatomic template. The maps were displayed using AFNI (http://afni.nimh.nih.gov/). We thresholded the maps to present only voxels with weight value above 30% of the maximum weight value. The red areas indicate higher weights for the positive class (i.e., 100% happy) and the blue areas higher weights for the negative class (i.e., neutral). HC = healthy comparison subjects; UD = currently depressed individuals with recurrent unipolar depression; BD = currently depressed individuals with bipolar I disorder.

Figure 4 reveals that the discriminating pattern between intense happy versus neutral faces was different for the three groups. HC showed a well-defined pattern of lateral prefrontal, visual, and parietal cortical regions discriminating neutral from intense happy faces. This pattern was less defined in UD, and even less in BD. These regions are important for visual attention and suggest that BD in particular did not use these regions to discriminate between neutral and intense happy stimuli. It should be emphasized, however, that pattern recognition approaches are multivariate techniques and that discrimination is based on the whole pattern rather than on individual/regional activities. It is therefore not possible to make local inferences about discriminating regions, as these can be interpreted only as a distributed discriminating pattern (see *Supplementary material* for discriminating regions for each classifier).

## Discussion

A critical challenge for psychiatry is to identify biological measures that may help to discriminate between bipolar and unipolar depression, given the frequent misdiagnosis of the former as the latter (1). Previous studies reported differential patterns of activity and effective connectivity to emotional faces, particularly happy faces, in UD and BD (3–6). The aim of the present study was to employ GPCs to examine the extent to which UD and BD differed in discrimination between patterns of neural activity to happy and to neutral face stimuli. Here, we used a two-level novel analytic strategy. In the first level, we used GPCs to discriminate patterns of neural activity elicited by different facial expressions within each group (HC, UD, and BD); that is, we computed the within-group classification accuracy for different stimulus contrasts and the predictive probabilities for each stimulus. In the second level, we used ANOVA and post-hoc tests to examine whether the predictive probabilities were significantly different between groups. We also performed the more conventional strategy of determining whether GPCs computed using the patterns of neural activity for each of three emotional faces (intense happy, mild happy, and neutral faces) could accurately discriminate between groups.

Our novel analytic strategy revealed the most interesting findings. Here, we showed that the predictive probability for intense happy faces was significantly higher in HC than BD, but *not* in HC versus UD. Importantly, the predictive probabilities were also *higher* in UD than in BD. As the predictive probability represents a measure of the classifier confidence about the class membership of the pattern being classified, these results indicate that the pattern of neural activity for intense happy faces was significantly less distinct from that for neutral faces in BD than in either HC or UD. One possible explanation for the lower level of discrimination of the pattern of neural activity to intense happy faces in BD was that there may have been greater variation in neural activity patterns to these stimuli in BD than in the other two groups. Another possibility is that there may have been greater overlap between the pattern of neural activity for intense happy faces and for neutral faces in BD than in the other two groups. We also showed that the predictive probabilities of whole-brain neural activity patterns to mild happy faces were significantly higher in HC versus BD and in HC versus UD. Our more conventional approach of examining the ability of the GPC to discriminate directly between groups also indicated that the best classification accuracy was obtained when directly discriminating between BD and UD using patterns of neural activity for mild happy faces, although this discrimination was at an accuracy level of 67%.

Our findings parallel other neuroimaging reports that indicated that BD and UD are distinguished by different patterns of neural activity to those for happy faces. For example, we previously reported abnormally elevated subcortical limbic activity to happy faces in bipolar disorder patients with subthreshold depression, but not in UD (4, 5), and differential patterns of orbitomedial prefrontal cortical–amygdala functional (effective) connectivity to happy faces in BD and UD (6). These findings suggest functional abnormalities in neural circuitry supporting positive emotional stimuli (happy faces) in BD that, in turn, may represent vulnerability to manic states and reflect biological processes that can distinguish BD from UD. Thus, BD and UD may be distinguishable from each other, as well as from HC, by fMRI activation, effective connectivity, and predictive probabilities of whole-brain patterns of neural responses to happy faces.

GPC can also provide a spatial map showing the most discriminating regions (i.e., the relative importance of each brain voxel for the discrimination boundary). The discrimination map should not, however, be interpreted as a representation of the statistical differences between classes but as a representation of the boundary between classes. Specifically, a high value in a particular voxel indicates a strong contribution to the discrimination boundary but does not necessarily imply greater activation in either condition. The most discriminating regions for discriminating between happy and neutral stimuli in HC included a pattern of lateral prefrontal, visual, and parietal cortical regions. These regions had the least weight for BD. In addition, the discriminating pattern for BD contained the most noise, suggesting that the spatial patterns were less consistent in BD.

A limitation of our study was that both depressed groups were taking medication, as would be expected, given the severity of depression in the patients recruited, and some BD and UD had a previous history of substance abuse. BD were taking more medication (indicated by greater medication load) than UD because of treatment of BD, but not UD, with mood stabilizers and antipsychotic medications. In within-group classification analyses, we therefore used a residual forming matrix (23) to extract the variance in the data explained by medication load and substance abuse before training the classifiers. This procedure is equivalent to dummy variable regression or analysis of covariance. In this way, we aimed to account for the potential confounding effects of medication and history of substance abuse in our within-group classification analyses. It is clearly difficult to completely control for potentially confounding effects of medication. One possibility would have been to include only unmedicated patients in the study. We would, however, suggest that this may have biased the study to examining individuals who are not representative of depressed populations, given that many depressed individuals require medication. As reviewed elsewhere in this issue (24), in fMRI studies, medication effects appear to minimally impact group differences, although they may be normalizing, thereby potentially minimizing rather than exaggerating differences among the groups. Another potential limitation was that, while there was no significant between-group difference in depression severity, with both groups having mean depression scores in the severe range of the 25-HDRS (19–22), UD had higher mean depression severity than BD. Additionally, some UD and BD had depression scores in the mild/moderate range on the day of scanning (i.e., scoring between 13 and 18 on the 25-HDRS). From the methodological point of view, the main limitation is the sample size. While the leave-one-out cross-validation framework is the recommended technique for evaluating classifier performance with small samples, due to its almost unbiased estimation of the true error rate, it has high variance for small sample sizes. Therefore, our results should be validated using independent and bigger samples. Future directions will be to replicate our results with larger sample sizes, ideally from different centers, in even more severely depressed individuals. Additionally, future studies employing GPC to help to classify individuals with either bipolar or unipolar depression could aim to include other types of neuroimaging data (e.g., resting state data) and potentially adding additional clinical information into the model.

To our knowledge, this study is the first to investigate the discriminability in whole-brain activity patterns elicited by positive emotional and neutral stimuli between unipolar and bipolar depression. Pattern recognition approaches have been used previously to discriminate between different brain states in healthy individuals (7–9) and also to discriminate between healthy and UD subjects (15, 16), based upon whole-brain patterns of activity in response to a specific stimulus (e.g., sad faces), task (e.g., verbal working memory), or a combination of emotional and affective processes (17). While our findings did not reveal robust findings regarding the ability of GPC to directly discriminate among groups, the key innovation of the present study was the use of a GPC to perform *within-group* brain classification analysis, and then to use the predictive probabilities of whole-brain patterns of neural activity to happy faces to discriminate between two groups of patients with highly overlapping symptoms. In clinical practice, it is critically important to have a test to identify, on the basis of an individual person’s task performance, the likely diagnosis. Our findings showed that GPCs predicted whole-brain activity in response to intense happy faces (presented alongside neutral faces) less confidently (i.e., with lower predictive probabilities) in BD than in HC and UD. A future application of this technique would therefore aim to discriminate between BD and UD on the basis of their individual whole-brain activity patterns elicited by positive emotional and neutral stimuli (e.g., using individual-level whole-brain predictive probability for intense happy faces).

Pattern recognition approaches have promising clinical utility as a potential methodology to discriminate not only between healthy and psychiatrically unwell individuals, but also among patients with different psychiatric illnesses. This approach can ultimately help to improve the diagnoses of those psychiatric illnesses that are often extremely difficult to accurately diagnose using current clinical criteria. These approaches may have wider future use in identifying abnormal patterns of neural activity in patient populations that predict subsequent response to different treatments, and inter-individual differences in specific information-processing domains that denote risk for future psychiatric illnesses.

## Supporting information

**Table S1.** Between-group accuracy

**Table S2.** Medication list

**Table S3.** HC group: most discriminative areas for 100% happy versus neutral

**Table S4.** UD group: most discriminative areas for 100% happy versus neutral

**Table S5.** BD group: most discriminative areas for 100% happy versus neutral
