# Supplementary material for: Pattern recognition analyses of brain activation elicited by happy and neutral faces in unipolar and bipolar depression
Source: Bipolar Disord. 2012 Jun;14(4):451–60. doi: 10.1111/j.1399-5618.2012.01019.x (PMC3510302; doi:10.1111/j.1399-5618.2012.01019.x)
Supplement: Supplementary file 1 [file bdi0014-0451-SD1.doc]

**Supplementary Material**

| **Table S1. Between-group accuracy** | | | | | | |
| --- | --- | --- | --- | --- | --- | --- |
| Groups | N per group | Task | Accuracy | TP | TN | p-value |
| BD × HC | 18 | 100% happy | 0.58 | 0.44 | 0.72 | 0.260 |
| BD × HC | 18 | 50% happy | 0.64 | 0.56 | 0.72 | 0.061 |
| BD × HC | 18 | Neutral | 0.56 | 0.56 | 0.56 | 0.455 |
| UD × HC | 18 | 100% happy | 0.53 | 0.50 | 0.56 | 0.444 |
| UD × HC | 18 | 50% happy | 0.61 | 0.50 | 0.72 | 0.117 |
| UD × HC | 18 | Neutral | 0.59 | 0.50 | 0.67 | 0.203 |
| BD × UD | 18 | 100% happy | 0.59 | 0.50 | 0.67 | 0.213 |
| **BD** × **UD** | **18** | **50% happy** | **0.67** | **0.72** | **0.61** | **0.018** |
| BD × UD | 18 | Neutral | 0.59 | 0.67 | 0.50 | 0.193 |

**Medication load**

We developed a strategy for measuring total medication load in BD (1, 2) by coding the dose of each antidepressant, mood-stabilizer, antipsychotic and anxiolytic (benzodiazepine) medication as absent (0), low (1), or high (2) dose. For antidepressants and mood stabilizers, we converted each medication into low- or high-dose groupings using a previously employed approach (3). Patients on levels 1 and 2 of these criteria were coded as low-dose, those with levels 3 and 4 as high-dose. We added a no-dose subtype for those not taking these medications. We converted antipsychotic doses into chlorpromazine dose equivalents, and coded as 0, 1, or 2, for no medication, chlorpromazine equivalents dose equal or below, or above, the mean effective daily dose (ED 50) of chlorpromazine as defined previously (4). Benzodiazepine anxiolytic dose was similarly coded as 0, 1, or 2, with reference to the midpoint of the Physician’s Desk Reference-recommended daily dose range for each medication. We generated a composite measure of total medication load, reflecting dose and variety of all different medications taken, by summing all individual medication codes for each medication category for each individual participant. BD had significantly greater medication load, reflecting greater number and dose of different psychotropic medications (see Table 1 and Table S2).

**Table S2. Medication list**

| **COHORT** | **MRI_DATE** | **MED_DATE** | **ML** | **MEDS_1** | **MEDS_2** | **MEDS_3** | **MEDS_4** | **MEDS_5** |
| --- | --- | --- | --- | --- | --- | --- | --- | --- |
| BD | 22-Jun-06 | 22-Jun-06 | 4 | CYMBALTA | QUETIAPINE | LAMICTAL | CLONAZEPAM |  |
| BD | 02-Aug-05 | 26-Aug-05 | 3 | ABILIFY | TOPAMAX | LORAZEPAM |  |  |
| BD | 18-Jul-05 | 18-Jul-05 | 2 | LITHIUM | LAMICTAL |  |  |  |
| BD | 03-May-05 | 22-Apr-05 | 5 | SEROQUEL | ABILIFY | LAMICTAL | WELLBUTRIN | LITHIUM |
| BD | 18-Nov-05 | 15-Nov-05 | 0 |  |  |  |  |  |
| BD | 18-Aug-06 | 15-Aug-06 | 2 | BUPROPION | VALPROIC ACID |  |  |  |
| BD | 28-Jul-06 | 20-Jul-06 | 3 | PAROXETINE | TRAZODONE | LAMICTAL |  |  |
| BD | 04-Aug-06 | 28-Jul-06 | 4 | VENLAFAXINE | VALPROIC ACID | LORAZEPAM | HALDOL |  |
| BD | 21-Jan-06 | 18-Jan-06 | 5 | LITHIUM | QUETIAPINE | RISPERIDONE | LORAZEPAM | TOPAMAX |
| BD | 27-Jan-06 | 27-Jan-06 | 3 | LAMICTAL | OLANZAPINE | QUETIAPINE |  |  |
| BD | 02-Dec-05 | 02-Dec-05 | 3 | LITHIUM | RISPERIDONE | ALPRAZOLAM |  |  |
| BD | 07-Sep-06 | 22-Aug-06 | 2 | SERTRALINE | QUETIAPINE |  |  |  |
| BD | 14-Dec-06 | 21-Nov-06 | 5 | VALPROIC ACID | LITHIUM | ABILIFY | CLONAZEPAM | TRAZODONE |
| BD | 11-Dec-07 | 03-Dec-07 | 2 | FLUOXETINE | ZIPRASIDONE |  |  |  |
| BD | 08-Feb-08 | 25-Jan-08 | 2 | EFFEXOR | ABILIFY |  |  |  |
| BD | 13-Mar-08 | 25-Feb-08 | 0 |  |  |  |  |  |
| BD | 24-Sep-08 | 02-Sep-08 | 1 | PAXIL |  |  |  |  |
| BD | 08-Aug-08 | 07-Aug-08 | 0 |  |  |  |  |  |
| BD | 25-Apr-08 | 18-Apr-08 | 3 | SEROQUEL | LORAZEPAM | CYMBALTA |  |  |
| UD | 14-Aug-08 | 04-Aug-08 | 1 | WELLBUTRIN |  |  |  |  |
| UD | 03-Jul-08 | 03-Jul-08 | 3 | PRISTIQ | KLONOPIN | PAXIL |  |  |
| UD | 12-May-08 | 16-Apr-08 | 0 |  |  |  |  |  |
| UD | 16-Nov-06 | 10-Nov-06 | 4 | VENLAFAXINE | OLANZAPINE | CLONAZEPAM | CYMBALTA |  |
| UD | 06-Jul-06 | 09-Jun-06 | 0 |  |  |  |  |  |
| UD | 28-Jan-06 | 28-Jan-06 | 1 | VENLAFAXINE |  |  |  |  |
| UD | 15-Sep-06 | 16-Aug-06 | 0 |  |  |  |  |  |
| UD | 06-Jun-08 | 05-Jun-08 | 1 | WELLBUTRIN |  |  |  |  |
| UD | 21-Feb-06 | 20-Feb-06 | 1 | SERTRALINE |  |  |  |  |
| UD | 19-Jan-07 | 19-Jan-07 | 2 | FLUOXETINE | TRAZADONE |  |  |  |
| UD | 19-Oct-07 | 18-Oct-07 | 1 | CELEXA |  |  |  |  |
| UD | 12-Oct-07 | 01-Oct-07 | 1 | ATIVAN |  |  |  |  |
| UD | 27-Nov-07 | 19-Nov-07 | 3 | WELLBUTRIN | LEXAPRO | XANAX |  |  |
| UD | 29-Feb-08 | 13-Feb-08 | 1 | AGOMELATINE |  |  |  |  |
| UD | 09-May-08 | 14-Apr-08 | 1 | VENLAFAXINE |  |  |  |  |
| UD | 10-Jul-08 | 24-Apr-08 | 3 | EFFEXOR | RITALIN | WELLBUTRIN |  |  |
| UD | 11-Jul-08 | 10-Jul-08 | 1 | LEXAPRO |  |  |  |  |
| UD | 25-Jul-08 | 11-Jul-08 | 2 | CELEXA | ATIVAN |  |  |  |

ML (medication load).

**Removing potentially confounding variables**

A residual forming matrix, normally applied to the data to remove confounds (5), can be applied directly to the covariance function. This is a computationally efficient approach that is equivalent to applying a regression model to remove the variance in the data explained by the potentially confounding variables. In general the residual forming matrix has the form **R** = (**I**-**CC**^+^), where **C**_m,k_ is a matrix with one confound per column pluss an extra column of ones to centre the data. m is the size of the kernel matrix and + is the pseudoinverse of **C**. We can remove confounds of the covariance function using the equation, **K***_new_* = (**R**^T^**DD**^T^**R**) = **R**^T^**KR**. There are some important assumptions when using this approach. First the covariate or confound variable is not affected by the class labels. Second, the regression slope between the signal measured and the covariate is the same for both classes.

**Permutation test**

Permutation testing was used to derive a p-value for the GPC accuracies. Here, we permuted each class’s labels 1000 times (i.e., each time randomly assigning class 1 and class 2 labels to each pattern of brain activation) and repeated the cross-validation procedure. We then counted the number of times the accuracy was higher than the one obtained for the real labels. Dividing this number by 1000 we derived a p-value for the classification accuracies.

**Gaussian Process Classifiers (GPC)**

Here we present a summary of Gaussian Process Classifiers, for a detailed description please see 6 and for an application of GP regression and classification to neuroimaging please see 7. Gaussian Process Classifiers are probabilistic classifications models derived from Gaussian process for regression. A Gaussian Process is a generalization of the Gaussian probability distribution for functions, i.e. it describes a Gaussian distribution over functions. A Gaussian process is completely specified by its mean function and covariance function $GP\sim N(m(\mathbf{x}),k(\mathbf{x}_{i},\mathbf{x}_{j}))$. GP models use the Bayesian learning framework to learn an input-output relationship (e.g. regression) based on a training data and then make predictions for new examples (i.e. testing data). In the regression case the predictions are made by $f(\mathbf{x})=\mathbf{x}^{T}\mathbf{w}$ and $y=f(\mathbf{x})+\varepsilon$:

where **w** is a vector of weights and $\varepsilon\sim N(0,\sigma_{n}^{2})$ is a Gaussian noise term. The model learning consists of placing a zero-mean GP prior over the function (or weights), and then using the Bayes’ rule to compute the posterior distribution evaluated at the training data.

$p(\mathbf{w}|\mathbf{X},\mathbf{y},\theta)=\frac{p(\mathbf{w}|\theta)p(\mathbf{y}|\mathbf{X},\mathbf{w},\theta)}{p(\mathbf{y}|\mathbf{X},\theta)}$ (1)

Here, the targets are collected in a vector, $\mathbf{y}=[y_{1},...,y_{m}]^{T}$, $p(\mathbf{w}|\theta)$ describes the prior over the weights, the likelihood is denoted by $p(\mathbf{y}|\mathbf{X},\mathbf{w},\theta)$ and $\theta$ is a vector of hyper-parameters. The denominator is called the marginal likelihood (or model evidence) and can be written as $p(\mathbf{y}|\mathbf{X},\theta)=\int p(\mathbf{y}|\mathbf{X},\mathbf{w})p(\mathbf{w})d\mathbf{w}$. To make predictions for a test case, we integrate (average) over all possible values for **w**, weighted by their posterior probability: $p(f*|D,\theta,\mathbf{x}*)=\int p(f*|\mathbf{w},\mathbf{x}*,\theta)p(\mathbf{w}|D,\theta)d\mathbf{w}$.

In GP regression, the likelihood and prior are both GPs, which means the posterior predictive distribution is also Gaussian and can be computed in closed form:

$$p(f*|D,\theta,\mathbf{x}*)\sim N(\mu,\sigma^{2})$$

$$\mu=\mathbf{k}*^{T}\mathbf{C}^{-1}\mathbf{y}$$

$\sigma^{2}=k(\mathbf{x}*,\mathbf{x}*)-\mathbf{k}*^{T}\mathbf{C}^{-1}\mathbf{k}*$ (2)

where $\mathbf{C}=\mathbf{K}+\sigma_{n}^{2}\mathbf{I}$. **K** is a kernel matrix describing the covariance between each data sample, i.e.,: $\mathbf{K}_{ij}=k(\mathbf{x}_{i},\mathbf{x}_{j},\theta)$. Similarly, $\mathbf{k}*=[k(\mathbf{x}_{1},\mathbf{x}*),...,k(\mathbf{x}_{m},\mathbf{x}*)]^{T}$ is a vector of covariances between the test (**x*)** and training data (**X)**. There are a number of possible forms for the covariance function (Rasmussen and Williams, 2006) but here we use a linear covariance function which is parameterised as:

(3)

*l* is a length-scale parameter that controls how far down any axis it is necessary to travel before samples become de-correlated (or equivalently, how quickly predictive variance grows with increasing distance from the data points). *b* is a bias term that accommodates the offset from zero.

Gaussian Process classification is an extension of the regression case. In case of classification we place a prior over a latent function$f(\mathbf{x})$ and then use a response function to map it to the unit interval in order to obtain a prior on the class probabilities. Here we use the cumulative Gaussian density (or probit likelihood) to perform this mapping.

(4)

In GP classification we also use Bayesian learning framework to compute the posterior distribution over the latent function. For binary classification we can write each likelihood term as $p(y_{i}|f_{i})=\Phi(y_{i}f_{i})$ (owing to the symmetry of the probit likelihood) and re-write Bayes rule as:

$p(\mathbf{f}|D,\theta)=\frac{N(\mathbf{f}|\mathbf{0},\mathbf{K})}{p(D|\theta)}\prod_{i=1}^{m} \Phi(y_{i}f_{i})$ (5)

Here, $\mathbf{f}=[f_{1},...,f_{m}]^{T}$ is the latent function values at training points, $N(\mathbf{f}|\mathbf{0},\mathbf{K})$ describes the prior over the latent function and we have factorized the likelihood over training samples (because the class labels are independent given the latent function).

As the likelihood is non-Gaussian the posterior is also non-Gaussian therefore the integral (5) is analytically intractable. To solve this problem one needs to use analytic approximation of the integral or solutions based on Monte Carlo sampling. In this paper we use the expectation propagation (EP) algorithm^8^ to approximate the non-Gaussian posterior. Predictions for new examples are divided in two steps: first we compute the distribution of the latent variable corresponding to the test example, and then we use this distribution over the latent function to produce a probabilistic prediction.

**Within-group discriminating regions**

In Tables S3-S5 we present the Tailarach coordinates of the clusters peaks of the discriminating maps, for each group for the contrast of intense happy vs. neutral. We used AFNI (http://afni.nimh.nih.gov/afni) for visualization purposes, i.e. to find clusters and to display the GPC weights as an image (Fig. 4). The clusters were extracted using the script 3dclust in AFNI. We then used the Talairach Client software to identify the corresponding regions.

From a pattern recognition perspective the discriminating maps (or spatial patterns) are only significant if the accuracy is above chance and significant, which means the spatial pattern can be used to classify new examples therefore it is consistent across subjects.

| **Table S3.**  **HC group: most discriminative areas for 100% Happy versus Neutral** | | | | |
| --- | --- | --- | --- | --- |
| x | y | z | Areas | Weights |
| -8.8 | -86 | -14.4 | Lingual gyrus | -21.274 |
| 0 | 52.8 | 12 | Medial frontal gyrus | 19.237 |
| 41.2 | -38.7 | 56.1 | Inferior parietal lobule | -18.219 |
| 0 | 14.4 | 38.4 | Cingulate gyrus | -14.803 |
| -53 | 11.5 | 29.6 | Inferior frontal gyrus | -15.577 |
| -32.4 | 23.3 | -2.6 | Insula | -15.478 |
| 44.2 | -59.4 | -26.1 | Tuber | -16.463 |
| 20.6 | -83 | 38.4 | Precuneus | 15.075 |
| -44.2 | -62.3 | 6.2 | Middle temporal gyrus | -13.908 |
| 29.4 | 17.4 | 3.2 | Claustrum | -14.441 |
| 11.8 | -0.3 | -20.2 | Parahippocampal gyrus | 23.068 |
| 2.9 | -50.5 | 32.6 | Precuneus | 12.987 |
| -44.2 | 44 | -11.4 | Middle frontal gyrus | -13.672 |
| -38.3 | -3.3 | 56.1 | Middle frontal gyrus | -17.629 |
| -58.9 | -24 | 15 | Postcentral gyrus | -11.411 |
| 2.9 | -50.5 | 0.3 | Culmen | 17.616 |

| **Table S4.**  **UD group: most discriminative areas for 100% Happy versus Neutral** | | | | |
| --- | --- | --- | --- | --- |
| x | y | z | Areas | Weight |
| 2.9 | -56.4 | 26.7 | Cingulate gyrus | 22.563 |
| -41.2 | 11.5 | 29.6 | Middle frontal gyrus | -19.929 |
| 0 | 17.4 | 41.4 | Cingulate gyrus | -25.413 |
| -8.8 | -38.7 | -14.4 | Culmen | 22.296 |
| 29.4 | 23.3 | -5.6 | Inferior frontal gyrus | -22.269 |
| 41.2 | 8.5 | -14.4 | Superior temporal gyrus | 19.845 |
| -26.5 | -65.3 | 35.5 | Precuneus | -19.092 |
| -35.3 | 20.3 | -8.5 | Inferior frontal gyrus | -22.196 |
| -2.9 | -38.7 | 0.3 | Culmen | -25.581 |

| **Table S5.**  **BD group: most discriminative areas for 100% Happy versus Neutral** | | | | |
| --- | --- | --- | --- | --- |
| x | y | z | Area | Weights |
| -2.9 | 29.2 | 6.2 | Anterior cingulate | 26.005 |
| 0 | 5.6 | 50.2 | Superior frontal gyrus | -27.145 |
| 55.9 | -26.9 | 38.4 | Postcentral gyrus | -17.727 |
| 0 | -53.5 | 32.6 | Precuneus | 15.769 |
| -2.9 | -15.1 | 38.4 | Cingulate gyrus | 17.557 |
| -17.7 | -74.1 | 47.3 | Precuneus | -15.481 |
| 8.8 | -47.6 | 0.3 | Culmen | 19.274 |
| -41.2 | 11.5 | 23.8 | Middle frontal gyrus | -16.783 |
| 38.3 | 14.4 | -2.6 | Insula | -14.033 |
| 2.9 | -71.2 | -2.6 | Lingual gyrus | 18.948 |
| -14.7 | -74.1 | 6.2 | Lingual gyrus | -14.019 |
| 44.2 | -26.9 | 6.2 | Superior temporal gyrus | -14.864 |
| 2.9 | -38.7 | -11.4 | Culmen | 23.453 |
| -50 | -62.3 | 26.7 | Middle temporal gyrus | 17.851 |
| -17.7 | -59.4 | -14.4 | Declive | -17.309 |
| 53 | -44.6 | 23.8 | Inferior parietal lobule | -15.825 |
| -29.4 | -80 | 20.8 | Middle occipital gyrus | -13.798 |
| -53 | -24 | 6.2 | Superior temporal gyrus | -12.675 |

**References**

**1.** Gilbert A, Mataix-Cols D, Almeida J et al. Brain structure and symptom dimension relationships in obsessive-compulsive disorder: a voxel-based morphometry study. J Affect Disord 2008; 109: 117-126.

**2.** Versace A, Almeida J, Hassel S et al. Elevated left and reduced right orbitomedial prefrontal fractional anisotropy in adults with bipolar disorder revealed by tract-based spatial statistics. Arch Gen Psychiatry 2008; 65: 1041-1052.

**3.** Sackeim H. The definition and meaning of treatment-resistant depression. J Clin Psychiatry 2001; 62 (Suppl. 16): 10-17.

**4.** Davis J, Chen N. Dose response and dose equivalence of antipsychotics. J Clin Psychopharmacol 2004; 24: 192-208.

**5.** Friston K, Chu C, Mourão-Miranda J et al. Bayesian decoding of brain images. Neuroimage 2008; 39: 181-205.

**6.** Rasmussen C, Williams CKI. Gaussian Processes for Machine Learning. Cambridge, MA: The MIT Press, 2006.

**7.** Marquand A, Howard M, Brammer M et al. Quantitative prediction of subjective pain intensity from whole-brain fMRI data using Gaussian processes. Neuroimage 2010; 49: 2178-2189.

**8.** Minka T. A Family of Algorithms for Approximate Bayesian Inference. PhD Thesis: Massachusetts Institute of Technology, 2001.
